# Identification of a Novel Glycolysis-Related Gene Signature for Predicting Breast Cancer Survival

**DOI:** 10.3389/fonc.2020.596087

**Published:** 2021-01-08

**Authors:** Dai Zhang, Yi Zheng, Si Yang, Yiche Li, Meng Wang, Jia Yao, Yujiao Deng, Na Li, Bajin Wei, Ying Wu, Yuyao Zhu, Hongtao Li, Zhijun Dai

**Affiliations:** ^1^Department of Breast Surgery, The First Affiliated Hospital, College of Medicine, Zhejiang University, Hangzhou, China; ^2^Department of Oncology, The Second Affiliated Hospital of Xi’an Jiaotong University, Xi’an, China; ^3^Breast Center Department, The Fourth Hospital of Hebei Medical University, Hebei Medical University, Shijiazhuang, China; ^4^Department of Breast Head and Neck surgery, The 3rd Affiliated Teaching Hospital of Xinjiang Medical University (Affiliated Tumor Hospital), Urumqi, China

**Keywords:** bioinformatics, breast cancer, glycolysis, prognostic signature, The Cancer Genome Atlas

## Abstract

To identify a glycolysis-related gene signature for the evaluation of prognosis in patients with breast cancer, we analyzed the data of a training set from TCGA database and four validation cohorts from the GEO and ICGC databases which included 1,632 patients with breast cancer. We conducted GSEA, univariate Cox regression, LASSO, and multiple Cox regression analysis. Finally, an 11*-*gene signature related to glycolysis for predicting survival in patients with breast cancer was developed. And Kaplan–Meier analysis and ROC analyses suggested that the signature showed a good prognostic ability for BC in the TCGA, ICGC, and GEO datasets. The analyses of univariate Cox regression and multivariate Cox regression revealed that it’s an important prognostic factor independent of multiple clinical features. Moreover, a prognostic nomogram, combining the gene signature and clinical characteristics of patients, was constructed. These findings provide insights into the identification of breast cancer patients with a poor prognosis.

## Introduction

Cancer is a global public health problem and the second most important cause of death in America (1). The global cancer burden is estimated every year by the American Cancer Society. According to the latest data report, the numbers of breast cancer (BC) cases and deaths estimated to occur in 2019 were 271,270 and 42,260, respectively (2). The high incidence and mortality of female BC remain a global health challenge, and the global burden is still increasing in several countries (3–5). Moreover, improvement of the overall clinical outcome of patients is crucial (6). Therefore, there is an urgent need to develop effective prognostic models for predicting the overall survival (OS) in patients with BC and for guiding clinical practice.

Metabolic reprogramming is a key hallmark of cancer (7, 8). Sufficient energy and metabolic intermediates for biosynthesis are the foundation of tumor cell initiation, proliferation and metastasis (9). Thus, many types of cancer are characterized by enhanced level of glycolysis and suppressed mitochondrial metabolism (7, 8, 10). Glycolysis might promote cancer cell survival by providing ATP and lactic acid (the main energy sources in cancer cells) (11). It has been reported that increased levels of glycolysis promoted the proliferation, invasion, and migration of certain cancer cells through activation of different signaling pathways and also enhanced drug resistance (10, 12–14). Therefore, tumor aerobic glycolysis has possible implications for prognosis judgment and cancer treatment (15, 16). Several studies have proven that the activity of cancer cells was significantly inhibited after glycolysis levels were decreased (17, 18). Studies have also examined the role of glycolysis in prediction of patient survival. For example, higher TCF7L2 expression predicted worse prognosis in pancreatic cancer (19). Four glycolysis-related genes (GRGs) (AGRN, AKR1A1, DDIT4, and HMMR) were identified as closely related to the clinical outcome in patients with lung adenocarcinoma (20). The glycolytic gene expression signatures based on nine (*CLDN9*, *B4GALT1*, *GMPPB*, *B4GALT4*, *AK4*, *CHST6*, *PC*, *GPC1*, and *SRD5A3*) and 10 biomarkers (*HK2*, *HK3*, *LDHA*, *PKM2*, *GAPDH*, *ENO1*, *LDHB*, *PKLR*, *ALDOB*, and *GALM*) predicted poor prognosis in patients with endometrial cancer (21) and glioblastoma patients (22), respectively. Although previous studies have investigated the role of GRGs and glycolysis in the development of BC (23–25), comprehensive investigations in this field are still needed.

This study aimed to evaluate the GRG expression in BC based on TCGA data and to study the association between GRG expression and BC survival. To this end, we primarily selected genes by conducting gene set enrichment analysis (GSEA). Many studies have focused on differentially expressed genes in tissues for the identification of biomarkers. However, some genes with important biological functions or connections among gene regulatory networks, gene functions, and characteristics are not differentially expressed and are often easily ignored. Since GSEA can scientifically screen genes based on the overall expression levels and data trends, it does not require significant differences in gene thresholds. This improves the statistical analysis of gene expression and biological significance (26).

Finally, an 11-GRG risk signature effectively predicting patient prognosis was constructed in our study. Furthermore, our gene-based model, as an independent prediction factor, could identify that patients with a high risk score had poorer prognoses than those in the low-risk score. Additionally, the prognosis performance of the risk model was significantly better than that of other clinical characteristics. In addition, it showed better performance in both training and testing datasets for predicting the clinical outcome in BC patients.

## Materials and Methods

### Data Collection

In total, 1,632 patients with BC were selected from five cohorts. The Cancer Genome Atlas (TCGA) cohorts included 1,057 records of patients with BC, whose expression profiles and clinical data were downloaded from the TCGA data portal (https://portal.gdc.cancer.gov/). The combined International Cancer Genomics Consortium (ICGC) cohort formed by the merger of Breast Cancer-FR and Breast Cancer-KR cohorts included 149 BC patients and the clinical information and expression profiles were obtained from ICGC database (http://dcc.icgc.org). Three Gene Expression Omnibus (GEO) cohorts were GSE42568, GSE7390, and GSE58812 datasets, which expression matrixes were obtained from the GEO database (https://www.ncbi.nlm.nih.gov/geo/). The GSE58812 and GSE42568 expression profile was based on the GPL570 platform and respectively contained 107 and 121 BC samples (27, 28). The GSE7390 expression profile was based on the GPL96 platform, which cohort included 198 BC samples (29). The patients from TCGA were defined as a training cohort, while the four datasets from the ICGC and GEO were used for external validation. In addition, we also extracted detailed clinical information of TCGA cohort as shown in **Table 1**: age, pathological stage, estrogen receptor (ER; positive or negative) status, progesterone receptor (PR; positive or negative) status, human epidermal growth factor 2 (HER2; positive or negative) status, adjuvant chemotherapies, and T/N/M stage. GRG sets were searched from the Molecular Signatures Database (MSigDB) (30).

**Table 1:** Clinic pathological characteristics of extracted patients with breast cancer.

Characteristic	Group	No. of cases (%)
Age (years)	<60	571 (54.02)
	≥60	485 (45.88)
	Unknown	1 (0.09)
Pathological stage	Stage I	181 (17.12)
	Stage II	599 (56.67)
	Stage III	237 (22.42)
	Stage IV	19 (1.80)
	Unknown	21 (1.99)
Pathological T	T1	278 (26.30)
	T2	607 (57.43)
	T3	132 (12.49)
	T4	37(3.50)
	Unknown	3 (0.28)
Pathological N	N0	500 (47.30)
	N1	351 (33.21)
	N2	119 (11.26)
	N3	72 (6.81)
	Unknown	15 (1.42)
Metastasis	M0	880(83.25)
	M1	21 (1.99)
	Unknown	156 (14.76)
ER	positive	774(73.23)
	negative	234(22.14)
	Unknown	49 (4.64)
PR	positive	673 (63.67)
	negative	333 (31.50)
	Unknown	51 (4.83)
HER2	positive	165 (15.61)
	negative	629 (59.51)
	Unknown	263 (24.88)
Adjuvant therapy	No	416 (39.36)
	Yes	534 (50.52)
	Unknown	107 (10.12)
Vital status	Alive	908 (85.90)
	Dead	149 (14.10)

ER, estrogen receptor; PR, progesterone receptor; HER2, human epidermal growth factor 2.

### Gene Set Enrichment Analysis

We used the GSEA (http://www.broadinstitute.org/gsea/index.jsp) to determine if the identified GRG sets had significant differences between the BC tissues and matched adjacent normal tissue (26). We use normalized P values of <0.05 to define statistical significance. The genes of the GRG sets which produced significant P value were collected for subsequent analysis.

### Construction and Evaluation of the 11-GRG Prediction Model

We normalized each gene from among the expression profiles using log2 transformation (20, 31, 32). We sequentially conducted univariate Cox, the least absolute shrinkage and selection operator (LASSO) regression using the R package “glmnet” (33, 34), and multivariate Cox regression analyses to identify the GRGs associated with BC prognosis and to construct a GRG-based prediction model (34–37). The risk score was calculated using the following formula: Risk score = $\Sigma_{\text{i}=1}^{\text{n}}coef*id$ (38). We performed the Kaplan–Meier survival analysis to assess the difference in survival between high and low risk score groups by using “survival” R package (39, 40). The time-dependent receiver operating characteristic (ROC) curve was used to assess the performance of the gene risk model and compare the prediction efficiency with clinical features using the “survivalROC” R package (39). We applied Cox regression analyses to assess the independent prognostic values of the signature and other clinical characteristics. To estimate the likelihood of survival, a nomogram was constructed based on the risk score and clinical features by using the R package of “rms” (41), which were analyzed using multivariate Cox regression analysis. And the prognostic ability of the nomogram was weighed by C-index, ROC, and calibration plots (41).

### Statistical Analysis

Differences among variables (risk score, age, tumor stage, T/N/M pathological stage, and ER, PR, and HER2 status) were tested using *t*-tests, non-parametric tests, or chi-square tests. We identified the alterations in selected genes from the cBioPortal website (http://www.cbioportal.org/). All statistical analyses were performed using R software (version 3.6.2) and R packages including “survivalROC”, “survival”, “glmnet” and “rms” (33, 34, 39, 40). P <0.05 was considered statistically different. All the scripts were uploaded at Github website (https://github.com/bioinformatics0/Glycolysis-BC).

## Results

### Initial Screening of Genes Using Gene Set Enrichment Analysis

**Figure 1:**
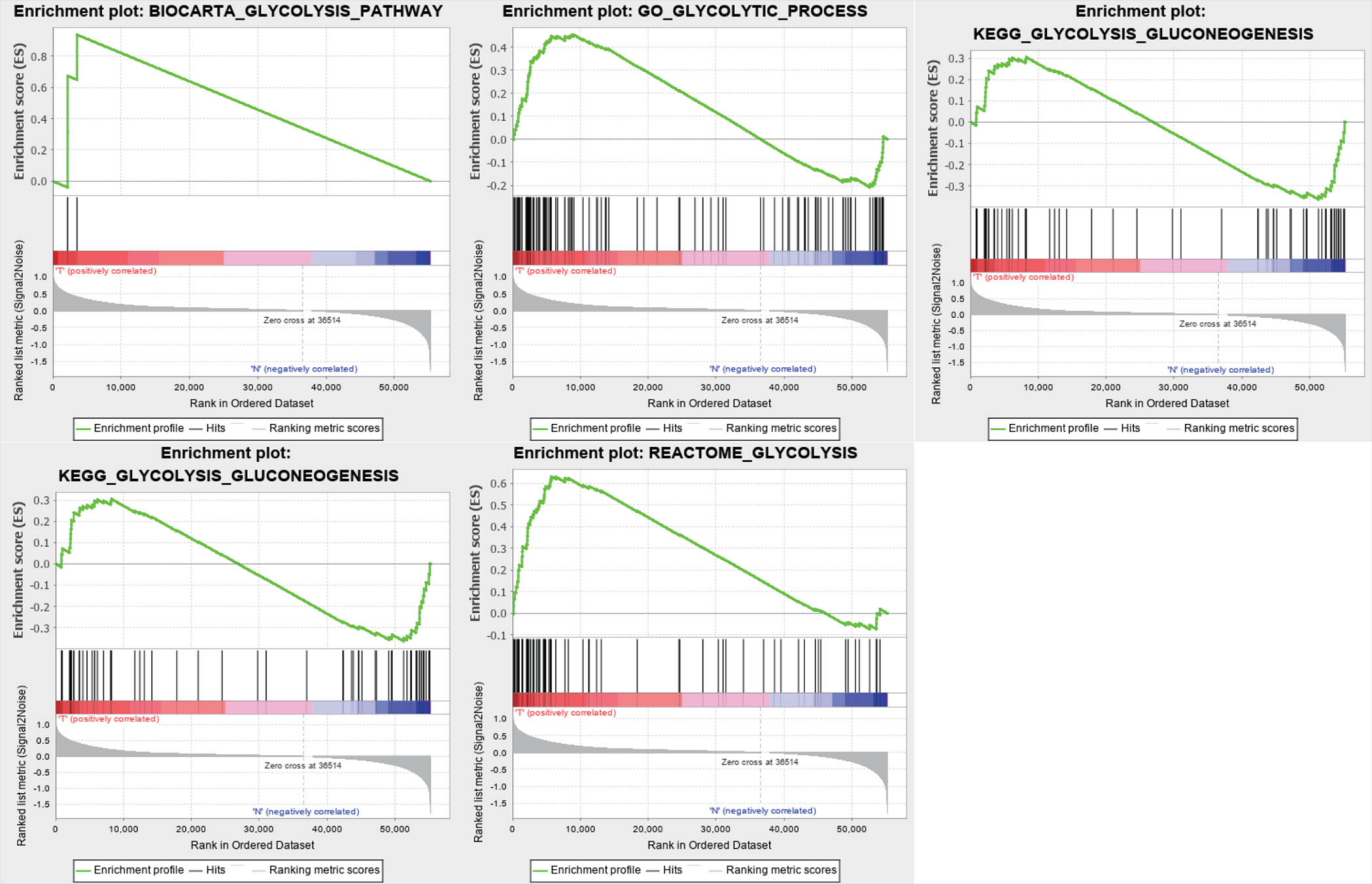
Enrichment plots of five gene sets which had significant difference between normal tissues and BC tissues by performing GSEA.

We obtained a dataset containing clinical information on 1,057 BC patients and 112 normal controls and data on the expression levels of 24,991 mRNAs from TCGA. Five glycolysis-related MSigDB version 6.2 gene sets were downloaded, and a total of 443 genes were obtained. We used the above data and GSEA to verify which gene sets had significant differences between the BC tissues and matched adjacent normal tissues. The results demonstrated four significantly enriched gene sets, with normalized P values <0.05, from the following pathways: BIOCARTA_GLYCOLYSIS_PATHWAY, GO_GLYCOLYTIC_PROCESS, HALLMARK_GLYCOLYSIS, and REACTOME_ GLYCOLYSIS (**Table 2**, **Figure 1**). The corresponding 381 genes from these four gene sets were selected for subsequent analysis.

**Table 2:** Gene sets enriched in breast cancer.

GS follow link to MSigDB	SIZE	ES	NES	NOM p-val	FDR q-val
BIOCARTA_GLYCOLYSIS_PATHWAY	3	0.94	1.54	0.0141	0.0141
GO_GLYCOLYTIC_PROCESS	106	0.45	1.63	0.0210	0.0210
HALLMARK_GLYCOLYSIS	200	0.58	2.06	0.0000	0.0000
KEGG_GLYCOLYSIS_GLUCONEOGENESIS	62	−0.36	−1.25	0.2064	0.2064
REACTOME_GLYCOLYSIS	72	0.63	2.05	0.0020	0.0020

### Construction and Evaluation of the Glycolysis-Related Risk Signature

**Figure 2:**
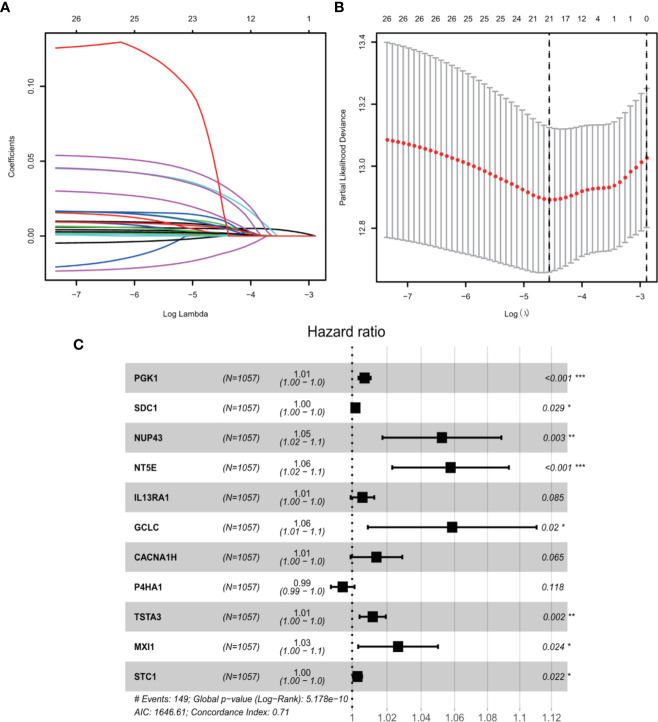
GRGs selection using the LASSO model and multivariable Cox model. **(A)** Ten-fold cross-validation for the coefficients of 326 GRGs in the LASSO model. **(B)** X-tile analysis of the 21 selected GRGs. **(C)** Forest plot illustrating the multivariable Cox model results of each gene in 11-GRG risk signature.

We conducted univariate Cox regression analysis to analyze 381 genes after GSEA. Finally, a total of 11 genes (*PGK1*, *SDC1*, *NUP43*, *NT5E*, *IL13RA1*, *GCLC*, *CACNA1H*, *P4HA1*, *TSTA3*, *MXI1*, and *STC1*) were significantly correlated with OS (adjusted P < 0.05) after the filtration using LASSO and multivariable Cox regression analyses (**Figure 2**). A gene-based prognostic model was established to evaluate the survival risk for each patient as follows: Risk score = 0.00710 × expression of *PGK1* + 0.00187 × expression of *SDC1* + 0.05107 × expression of *NUP43* + 0.05599 × expression of *NT5E* + 0.00587 × expression of *IL13RA1* + 0.05692 × expression of *GCLC* + 0.01385 × expression of *CACNA1H* + (-0.00535) × expression of *P4HA1* + 0.011698 × expression of *TSTA3* + 0.026129 × expression of *MXI1* + 0.00305 × expression of *STC1*. We then analyzed the mutational status of these 11 selected genes in TCGA BC samples in the cBioPortal database. **Figure S1A** shows the alterations in 11 genes. We also performed differential analysis of the expression of 11 genes in adjacent normal and BC tissues. Eleven genes were all significantly upregulated in tumor tissues (P < 0.05, **Figure S1B**).

**Figure 3:**
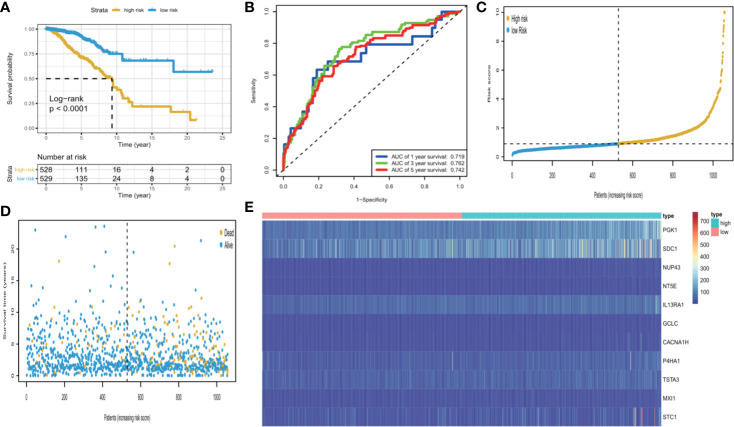
KM survival analysis, risk score assessment by the GRG‐related gene signature and time-dependent ROC curves in the TCGA cohort. **(A)** KM survival analysis of high‐ and low‐risk samples. **(B)** ROC curve for overall survival of the training set. The AUC was assessed at 1, 3, and 5 years. **(C)** Risk score distribution, **(D)**, survival status, and **(E)**. Eleven GRGs expression patterns for patients in high- and low-risk groups by the 11-GRG signature.

**Figure 4:**
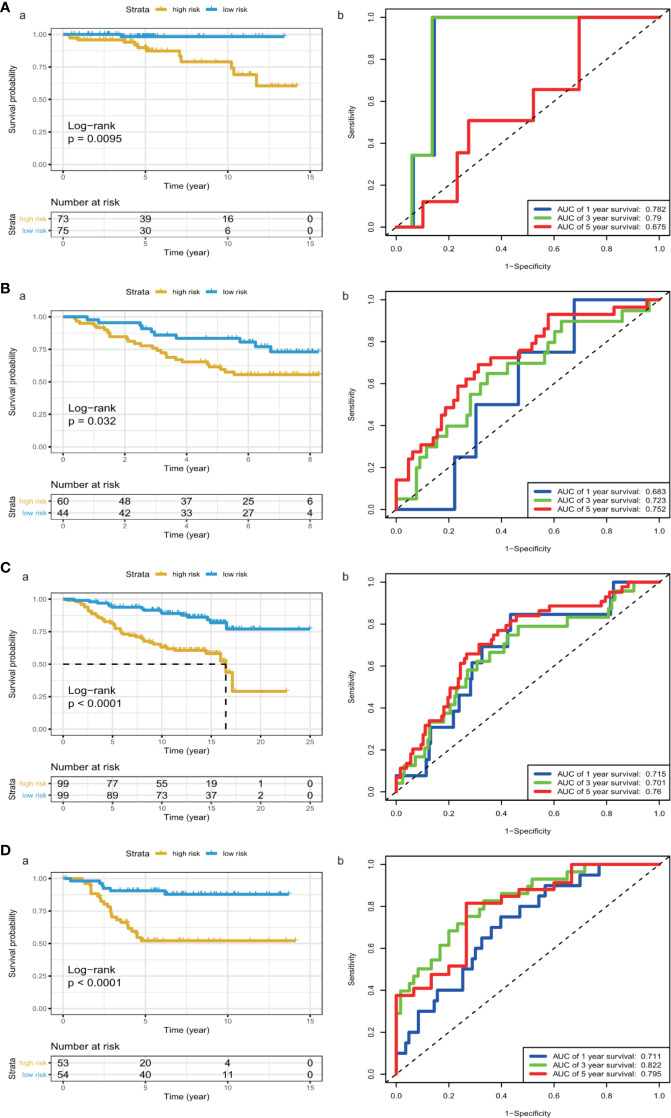
KM survival analysis and time-dependent ROC curves in the ICGC and GEO cohorts. **(A)** (ICGC), **(B)** a (GSE42568), **(C)** a (GSE7390), **(D)** a (GSE58812), Kaplan–Meier analysis with two-sided log-rank test was performed to estimate the differences in OS between the low-risk and high-risk group patients. **(A)** b (ICGC), **(B)** b (GSE42568), **(C)** b (GSE7390), **(D)** b (GSE58812), 1-, 3- and 5-year ROC curves of the 8-GRG signature were used to demonstrate the sensitivity and specificity in predicting the OS of BC patients.

We calculated each patient’s risk scores in the training set based on the 11-gene signature. Patients with a high-risk score had a higher mortality rate than those with a low-risk score (P < 0.0001, log-rank test) (**Figure 3A**). The area under the curve (AUC) values for 1-, 3-, and 5-year OS, were 0.719, 0.762, and 0.742, respectively (**Figure 3B**). **Figures 3C, D** show the risk scores rank distribution and survival status in BC patients in the training set. The expression patterns of 11 GRGs in high/low risk groups are shown in the heatmap (**Figure 3E**). To assess the robustness of the 11-GRG signature, we assessed its performance using validation cohorts from the ICGC and GEO databases. Similar to that in the previous analysis, the patients in the high-risk subgroup had poorer survival than those in the low-risk group (P < 0.05; **Figures 4Aa–Da**). The 1-, 3-, and 5-year AUC values were 0.782, 0.79, and 0.675 in the ICGC cohort (**Figure 4Ab**), and 0.683, 0.723, and 0.752 in the GSE42568 cohort, respectively (**Figure 4Bb**). The AUC for OS was 0.715 at 1 year, 0.701 at 3 year and 0.76 at 5 year in the GSE7390 cohort (**Figure 4Cb**), and 0.711 at 1 year, 0.822 at 3 year, and 0.795 at 5 year in the GSE58812 cohort (**Figure 4Db**).

### Establishment and Assessment of a Nomogram

**Figure 5:**
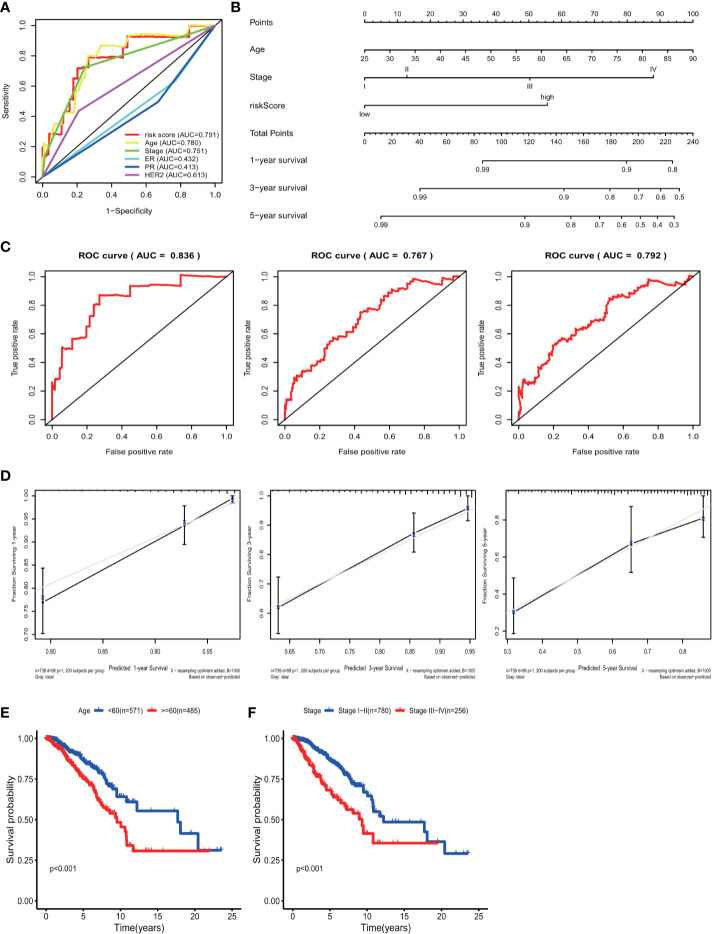
ROC curve with respect to clinical features and risk model, nomogram and Kaplan–Meier survival analysis for BC patients with clinical features: **(A)** Time-dependent ROC curve with respect to single clinical features and risk model. **(B)** The nomogram for predicting probabilities of BC patients overall survival. **(C)** 1-, 3- and 5-year ROC curves for the nomogram. **(D)** The 1-, 3- and 5-year nomogram calibration curves, respectively. Kaplan–Meier survival analysis for BC patients with different clinical features that can predict patient survival (**E**, Age, **F**, Stage).

Univariate analyses were performed to examine the prognostic values of several clinicopathological features (age, pathological stage, ER, PR, and HER2). Consequently, the 11-GRG risk signature correlated with OS (hazard ration [HR] = 1.178; 95% confidence interval [CI], 1.128−1.231, P < 0.001) (**Table 3**). And age >60, (HR = 1.047; 95% CI, 1.030–1.064, P < 0.001), high pathological stage (III/IV) (HR = 2.022; 95% CI, 1.541−2.654, P < 0.001) were also risk factors for BC. Furthermore, after the multivariate analyses, the results showed that risk score (HR = 1.136; 95% CI, 1.083−1.191), age (HR = 1.047; 95% CI, 1.030–1.065), and stage (HR = 1.986; 95% CI, 1.522−2.591) remained independent prognostic factors with an adjusted P value <0.001. In addition, the ROC analysis revealed that the sensitivity and specificity of the 11-gene signature were greater than those of the other clinicopathological features (**Figure 5A**). Additionally, the gene risk model was proven to be a competitive prognostic factor for BC survival prediction. These results suggested that the signature can be a promising prognostic indicator for predicting OS in patients with BC. To develop a quantitative method that can predict the OS of patients with BC, a nomogram was constructed. The predictors included risk score, age, and tumor stage which produced significant P value in multivariate Cox analysis (**Figure 5B**). The result of C-index (0.812), AUC (1-year, 0.836; 3-year, 0.767 and 5-year, 0.792) and calibration plot showed the nomogram predicts with high accuracy (**Figures 5C, D****)**.

**Table 3:** The risk score generated from the 11-GRG signature as an independent indicator according to Cox proportional hazards regression model.

Variable	Univariate analysis		Multivariate analysis	
	HR (95%CI)	*P*-value	HR (95%CI)	*P*-value
**Age (>60/≤60 years)**	1.047 (1.030–1.064)	<0.001	1.047 (1.030–1.065)	<0.001
**Pathological stage (I/II/III/IV)**	2.022(1.541–2.654)	<0.001	1.986(1.522–2.591)	<0.001
**ER (Negative/Positive)**	0.736(0.474–1.142)	0.171	0.846(0.434–1.651)	0.624
**PR (Negative/Positive)**	0.782(0.520–1.175)	0.236	0.803(0.435–1.483)	0.484
**HER2 (Negative/Positive)**	1.240(0.764–2.014)	0.384	1.080(0.661–1.765)	0.759
**Eleven-GRG risk scores (H/L)**	1.178(1.128–1.231)	<0.001	1.136(1.083–1.191)	<0.001

GRG, glycolysis-related gene; ER, estrogen receptor; PR, progesterone receptor; HER2, human epidermal growth factor 2. H, High; L, low.

### Data Stratification Analyses

**Figure 6:**
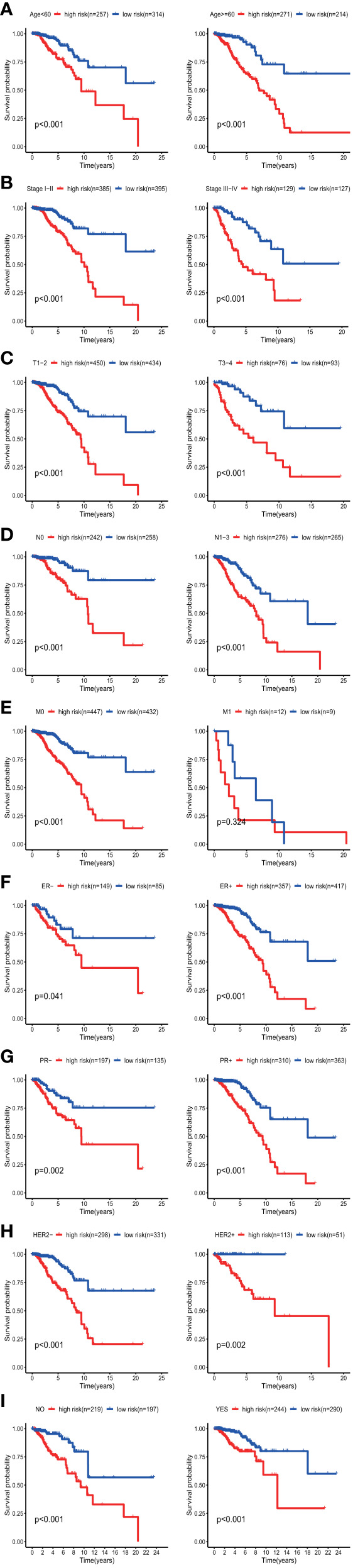
KM survival subgroup analysis of all patients with BC according to the GRG‐related gene signature stratified by clinical characteristics. **(A)** Age <60 y, Age >=60 y. **(B)** Early stage (stages I–II), Late stage (stages III–IV). C, T1-2, T3–4. **(D)** Lymph node-negative patients, Lymph node-positive patients. **(E)** Patients without distal metastasis, patients with distal metastasis. **(F)** ER-negative patients, ER-positive patients. **(G)** PR-negative patients, PR-positive patients. **(H)** HER2-negative patients, HER2-positive patients. **(I)** No adjuvant therapy, adjuvant therapy. GRGs, glycolysis-related genes; BC, breast cancer; ER, estrogen receptor; PR, progesterone receptor; HER2, human epidermal growth factor 2.

The results of the univariate Cox regression analysis of OS showed that age and stage could effectively predict survival in BC patients. The Kaplan–Meier curves revealed that the clinical features and results were consistent. BC patients who were older than 60 years and had stages III–IV disease were associated with poor prognosis (**Figures 5E, F****)**. In the TCGA cohort, subgroup analyses were conducted based on the clinicopathological variables (age, tumor stage, T/M/N stage, ER status, PR status, HER2 status and adjuvant chemotherapies). According to the Kaplan–Meier curves, in patients with BC who were stratified by age, tumor stage, T/N stage, ER status, PR status, HER2 status and adjuvant chemotherapies (No/Yes), the risk score remained a stable prognostic factor (**Figures 6A–D, F–I**). Nevertheless, the risk score played different roles in the subgroups or in patients stratified by metastasis stage. Patients in the high-risk group had a significantly shorter OS than those in the low-risk group in the subgroup of patients without distal metastasis (P < 0.001), while no significant difference was observed between the two groups with distal metastasis (P = 0.324) (**Figure 6E**). This result indicated that the risk model had better predictive value for clinical outcomes in BC patients without metastasis than in those with distal metastasis and more evidence and larger cohorts are necessary for further validation.

### Comparison With Other Prognostic Signatures

A comparison of our nomogram and signature with other known prognostic hallmarks was performed. In order to exclude the impact of heterogeneity, all of these hallmarks that were developed based on TCGA database were included. Considering that our research is based on all types of BC and total TCGA BC cohort was used as the training set, so we further excluded the studies with the model construction for specific BC subtype (42–44) and studies which TCGA cohort was randomly divided into training and testing sets (45, 46). Finally, 15 related prognostic signatures were included to compare with our gene signature and nomogram (**Table 4**). The AUCs of the signature and the nomogram in our study at 1-, 3-, and 5-years were 0.719, 0.762, 0.742 and 0.836, 0.767, 0.792 respectively. **Table 4** showed that the AUCs of four prognostic signature including 12 stemness-related lncRNA signature (0.813 at 5 years) (47), 11 immune-related lncRNA signature (0.836 at 5 years) (52), 27 immune-related gene signature (0.844 at 5 years) (54) and four methylated gene signature (0.791 at 5 years) (61) were distinctly higher than that of other biomarkers. Moreover, our signature also performed better in the prediction of BC patients’ OS than the signature based on the hallmarks related to autophagy (48), tumor microenvironment (immune, stromal, and proliferation) (49), tumor mutation burden (50), hypoxia (51), DNA repair (55), lncRNA (56) and miRNA (57, 58). The larger the AUC value of the biomarkers, the better the predictive ability of the hallmarks. This clearly shows that our nomogram and GRG signature are superior to other models after the four high-quality models in predicting the OS of BC patients.

**Table 4:** The area under the ROC curve (AUC) show the sensitivity and specificity of the known signatures in predicting the prognosis of BC patients.

Author	Year	Gene Signature	AUC for OS
Li X, et al (47)	2020	12 stemness-related lncRNA signature	0.813 (5-year)
Lin Q, et al (48)	2020	12 autophagy-related gene signature	0.739(1-year), 0.727(3-year), 0.742(5-year),
Wang J, et al (49)	2020	four ISP gene signature	0.742 (5-year)
Wang F, et al (50)	2020	six gene TMB-based signature	0.705 (5-year)
Wang J, et al (51)	2020	14-gene hypoxia−related signature	0.728 (1-year), 0.726 (3-year), 0.736 (5-year)
Shen Y, et al (52)	2020	11 immune-related lncRNA signature	0.836 (5-year)
Xu H, et al (53)	2020	eight immune-related gene signature	0.753 (3-year), 0.72 (5-year)
Zhao Y, et al (54)	2020	27 immune-related gene signature	0.844 (5-year)
Zhang D, et al (55)	2020	eight DNA repair–related gene signature	0.708 (3-year), 0.704 (5-year)
Sun M, et al (56)	2019	eight lncRNA signature	0.725 (1-year), 0.727 (3-year), 0.721 (5-year)
Kawaguchi, et al (57)	2019	three miRNA signature	0.71 (5-year)
Lai J, et al (58)	2019	six microRNA model	0.705 (3-year), 0.701 (5-year)
Liu L, et al (59)	2019	seven RNA signature	0.705 (5-year)
Tao C, et al (60)	2019	seven DNA methylation site signature	0.704 (5-year)
Feng L, et al (61)	2018	four methylated gene signature	0.791 (5-year)

OS, overall survival; ISP, immune, stromal, and proliferation; TMB, tumor mutation burden.

## Discussion

BC is the most common cause of cancer-related mortality among malignancies and women worldwide (6, 62). It is difficult to predict prognosis in BC due to its phenotypic and molecular diversity. The application of prognostic models is useful for guiding clinical decisions and is essential for precision medicine. Subtype identification, risk stratification, and characterization of the underlying mechanisms are critical for the improvement of the existing treatment methods, development of more precise and personalized therapies, and prolongation of survival time. Glycolysis is a multi-step enzymatic reaction and is considered to be the root of the development and progression of cancer (63). Since an increasing number of studies have identified prognostic markers of GRGs, a GRG-based risk signature for predicting the survival in BC patients must be established to improve the accuracy in prognosis.

GSEA is a method for evaluating whole-genome expression profile chip data, which can integrate data from different levels and sources. In the present study, GSEA was conducted using the data on mRNA expression profiles in the 1,096 BC patients. Four gene sets with P values <0.05 exhibited significant differences and were chosen for subsequent analyses. Univariate, multivariate Cox, and LASSO regression analyses were performed to identify 11 prognostic genes for BC patients. Based on the 11 most valuable biomarkers, we developed and verified an effective model to predict clinical outcomes in BC patients. Survival analysis showed distinctly different prognoses between high- and low-risk BC patients. The model was also verified in the GEO and ICGC datasets, demonstrating favorable clinical predictive ability. In addition, the prediction model for BC patients could act as an independent prognostic tool through multivariate Cox analyses. We also found that patients with higher risk scores in our prediction model tended to be older, have advanced stage disease, and a poorer prognosis. The prediction model in our study had similar or better clinical application potential compared to traditional clinical factors. Moreover, we integrated the prediction model and clinical characteristics to establish a novel nomogram. The nomogram took advantage of the complementary values of clinical characteristics and the prediction model and provided superior estimation of OS. The result showed that C-index, ROC and calibration plot performed well in our study. Additionally, the gene signature could further stratify clinically defined groups of patients (*e.g.*, groups stratified according to age, stage, T/N/M stage, ER status, PR status, HER2 status and adjuvant chemotherapies) into subgroups with different survival outcomes. The risk model could effectively predict the prognosis of patients with BC in all subgroups, but it could not be applied to the subgroup of BC patients with distal metastasis. The underlying mechanisms of this result should be explored in depth in the future. The results showed that the calculation of risk scores has great prognostic significance for BC patients. This not only increases the means of predicting the prognosis but can also help clinicians to choose more suitable treatment options for patients.

Chemotherapy is still an important way of cancer treatment. Chemotherapy drugs have an oxygen-dependent effect on the killing of tumor cells, most of which kill cells by oxidizing free radicals and reactive oxygen species in cells. Hypoxia can significantly reduce the efficiency of chemotherapy (64). The Warburg effect is aerobic glycolysis in cancer cells, which has been found to be involved in chemotherapy resistance in various types of human cancers (65, 66). The Warburg effect promotes epigenetic and genetic changes leading to the occurrence of multiple new cell phenotypes including the existence of drug resistance cells (67). To confirm whether our signature can provide an effective prediction method for the prognosis of patients receiving adjuvant chemotherapy, we conducted a subgroup analysis and the results showed the risk model could effectively predict the prognosis of patients with BC in both receiving and not receiving adjuvant chemotherapy groups. This also shows the extensive clinical application of our model.

To further explore the predictive ability of our nomogram, a comparison was performed among several significant molecular signatures which were employed for predicting OS of BC patients. The studies (47, 48, 52, 54, 61) we included were that the model was built based on the entire TCGA cohort and involved all types of breast cancer, not a certain subtype. The final results showed that our signature and another four prognostic signature including 12 stemness-related lncRNA signature (47), 11 immune-related lncRNA signature (52), 27 immune-related gene signature (54) and four methylated gene signature (61) performed better in the prediction of BC patients’ OS than the signature based on the hallmarks related to autophagy (48), tumor microenvironment (immune, stromal, and proliferation) (49), tumor mutation burden (50), hypoxia (51), DNA repair (55), lncRNA (56) and miRNA (57, 58). Considering that the clinical application cost of our model may be lower than that of the two gene models [12 stemness-related lncRNA signature (47) and 27 immune-related gene signature (54)] and glycolysis is closely related to the prognosis of BC, our signature may be necessary to enrich the clinical prediction methods. What’s more, the AUC of the nomogram is greater than that of the signature in our study, suggesting that the combination of the risk score with clinical factors is more promising than the methylation signature alone in predicting the OS of BC patients.

The 11 GRGs identified in this study included *PGK1*, *SDC1*, *NUP43*, *NT5E*, *IL13RA1*, *GCLC*, *CACNA1H*, *P4HA1*, *TSTA3*, *MXI1*, and *STC1*. Of these genes, *PGK1* (phosphoglycerate kinase 1) has been identified to promote BC progression and metastases *via* forming a positive feed-forward loop with HIF-1α. High *PGK1* expression predicted poor prognosis in BC (63). *SDC1* (syndecan-1), a heparin cell surface proteoglycan, can act as a co-receptor for growth factors and chemokines (68). High expression of *SDC1* has been identified in BC tissues as associated with an aggressive phenotype and poor clinical behavior (69). Nup43 (nucleoporin 43 kDa) is a stable component of the Nup107 160 complex, which is localized at kinetochores in mitosis and regulates mitotic progression and chromosome segregation (70). Higher expression of *NUP43* is often accompanied by DNA amplification and is related to poor OS in luminal A and HER2+ BC (71). *NT5E* (ecto-5-nucleotidase), also designated *CD73*, is a promising prognostic factor, and its high expression was significantly related to lymph node metastases in BC patients (72). A study reported that the interactions between interleukin-13 and interleukin-13 receptor alpha 1 (IL13RA1) contributed to cancer cell growth and metastasis, and IL13RA1 expression was associated with poor prognosis in invasive BC patients (73). Collagen prolyl 4-hydroxylase alpha 1 (P4HA1) is the major isoform in most cell types and tissues, and it can also enhance the activity of most prolyl 4-hydroxylases (74). During the development of BC, *P4HA1* expression is induced (75). When the *P4HA*/*HIF*-*1* axis is activated, the cancer cell stemness is enhanced, while the levels of oxidative phosphorylation and reactive oxygen species are reduced (76). The malignant transformation of cells and tumor development were promoted by abnormal glycosylation, which depends on *TSTA3* gene function (tissue-specific transplantation antigen P35B) (77). The survival rates in BC patients with a higher expression of *TSTA3* were lower (78). MYC-associated protein X interactor-1 (MXI1) is an antagonist of the oncogenic MYC protein, and the deletion of the *MXI1* gene causes many kinds of human cancers (79). The low expression of *MXI1* was related to poor prognosis in BC patients (80). Stanniocalcin-1 (STC1) is a secreted glycoprotein, and its high expression levels were associated with tumor growth and metastasis in BC (81). However, other genes (*GCLC* and *CACNA1H*) were identified for the first time to have prognostic value in BC patients. Deeper investigations of the biological functions of these genes in BC are warranted.

To our knowledge, our study is the first one to identify and comprehensively analyze prognostic GRGs for the prediction of survival in BC patients by evaluating the data from the public TCGA database. Moreover, a novel risk signature based on 11 GRGs was identified and verified. This signature can be used as a screening tool for patients at high risk of developing BC and to stratify patients to increase the effectiveness of targeted therapy. Additionally, we successfully established a GRG-related nomogram combining clinical factors and molecular markers to predict the OS of BC patients with in an effective quantitative approach. We also analyzed the mutational status of the nine selected genes in the cBioPortal database. Our research not only allowed to better understand the genetics of BC, but also had significance for guiding future research.

There are some limitations in our study. First, it was a retrospective study, and all BC patients were identified from public databases. Second, large-scale multicenter cohorts are necessary to validate the predictive performance of our model and to evaluate its clinical applicability for better management of BC. Furthermore, future basic experiments in our hospital will be required to conduct to verify our findings and elucidate the functional roles of GRGs involved in the initiation and development of BC. Moreover, the gene signature may be more effective to predict survival in BC patients without distal metastasis, and its prognostic role warrants further evaluation.

## Conclusion

We constructed a valid, innovative, and reliable 11-GRGs prognostic model (*PGK1*, *SDC1*, *NUP43*, *NT5E*, *IL13RA1*, *GCLC*, *CACNA1H*, *P4HA1*, *TSTA3*, *MXI1*, and *STC1*) to predict BC patient outcomes. Our signature was an independent and important risk factor for BC. Furthermore, a nomogram combining the prediction model and clinical factors was constructed, which could be a useful tool to predict prognosis and guide clinical practice.

## Data Availability Statement

The datasets generated and analyzed during the current study are available in the TCGA (http://cancergenome.nih.gov/abouttcga), GEO (https://www.ncbi.nlm.nih.gov/geo/) ICGC (http://dcc.icgc.org) and cBioPortal (http://www.cbioportal.org) databases. Accession number(s) can be found in the article/**Supplementary Material**.

## Ethics Statement

The studies involving human participants were reviewed and approved by the Institutional Review Board of the First Affiliated Hospital of Zhejiang University in Zhejiang Province (Hangzhou, China). The patients/participants provided their written informed consent to participate in this study. Written informed consent was obtained from the individual(s) for the publication of any potentially identifiable images or data included in this article.

## Author Contributions

DZ and YiZ collected and analyzed the data, and wrote the manuscript. SY, YL, MW, and JY analyzed the data and reviewed the manuscript. YD, NL, BW, YuZ, and YW participated in analyzing the data. DZ, YiZ, SY, and YL participated in the preparation of the figures and tables and interpretation of data for the work. ZD and HL designed the work and revised the manuscript. All authors contributed to the article and approved the submitted version.

## Conflict of Interest

The authors declare that the research was conducted in the absence of any commercial or financial relationships that could be construed as a potential conflict of interest.

## Supplementary Material

Supplementary Figure 1 Identification of GRGs related to patients’ survival. (A) Selected genes’ alteration in TCGA samples. (B) Different expression of eleven selected genes (*P < 0.05, **P < 0.01 and ***P < 0.001).

## Abbreviations

BC, Breast cancer; ER, Estrogen receptor; GEO, Gene Expression Omnibus; GRG, Glycolysis-related gene; GSEA, Gene set enrichment analysis; ICGC, International Cancer Genomics Consortium; LASSO, Least absolute shrinkage and selection operator; OS: Overall survival; PR, Progesterone receptor; ROC, Receiver operating characteristic; TCGA, The Cancer Genome Atlas.
