# Supplementary figures and images for: Identification of a Novel Glycolysis-Related Gene Signature for Predicting Breast Cancer Survival
Source: Front Oncol. 2021 Jan 8;10:596087. doi: 10.3389/fonc.2020.596087 (PMC7821871; doi:10.3389/fonc.2020.596087)

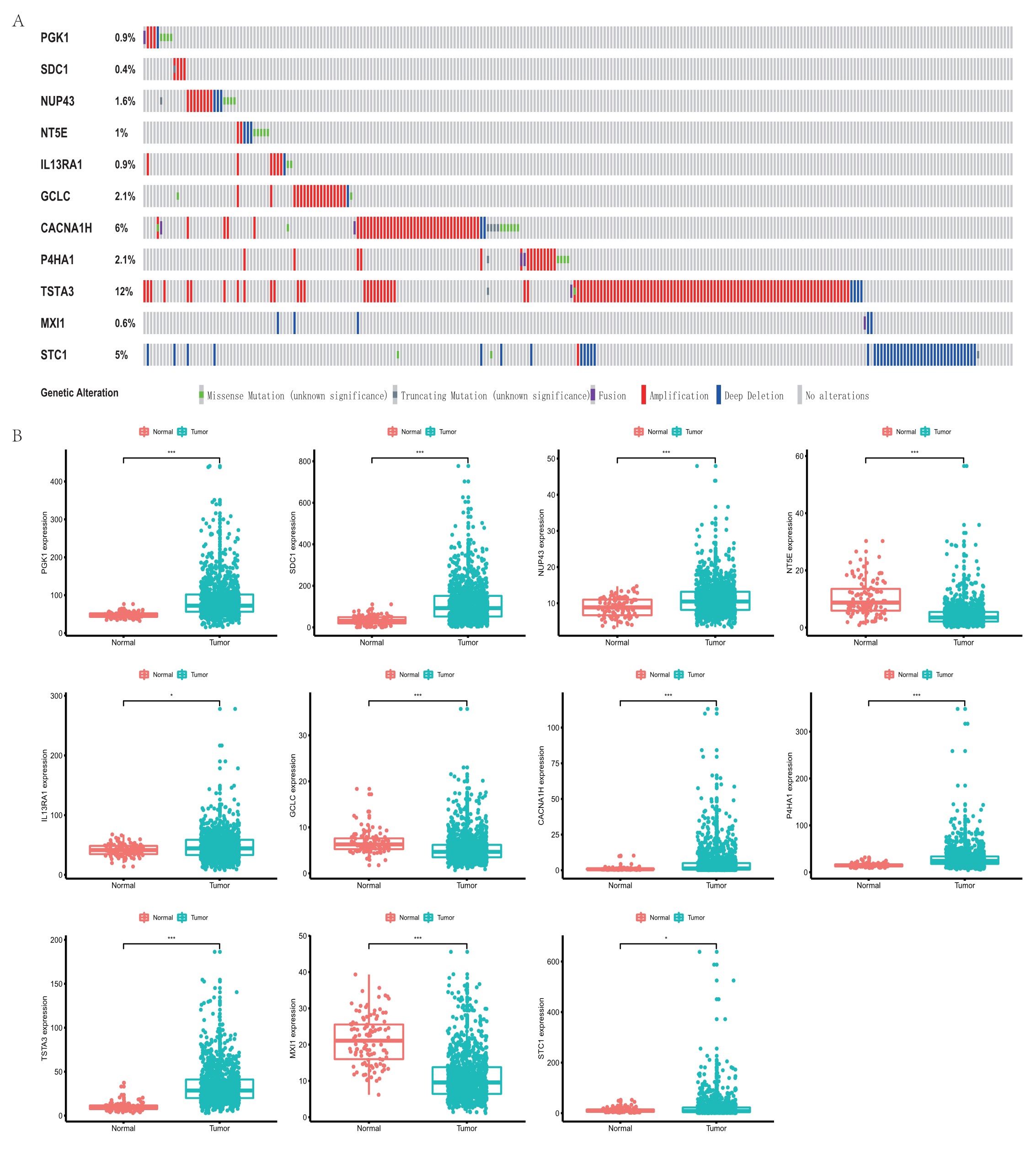

Supplement: Supplementary Figure 1 — Identification of GRGs related to patients’ survival. (A) Selected genes’ alteration in TCGA samples. (B) Different expression of eleven selected genes (*P < 0.05, **P < 0.01 and ***P < 0.001). [file Image_1.tiff]
